# Quality of life and level of satisfaction with pharmacotherapeutic follow-up in a transgender health center in Brazil

**DOI:** 10.1038/s41598-024-54737-y

**Published:** 2024-02-21

**Authors:** Carla Maria Lima Silva, Luiz Eduardo Oliveira Matos, Andressa Ribeiro Sassaqui, Alfredo Dias de Oliveira Filho, Chiara Erminia da Rocha, Giselle de Carvalho Brito

**Affiliations:** 1 Graduate Program in Pharmaceutical Sciences, Federal University of Sergipe, São Cristóvão, Sergipe Brazil; 2 Multiprofessional Residency in Hospital Health Care, University Hospital of Lagarto, Lagarto, Sergipe Brazil; 3 Graduate Program in Applied Health Sciences, Federal University of Sergipe, Governador Marcelo Déda Avenue, São José District, Lagarto, Sergipe 49400-000 Brazil; 4 Department of Pharmacy, Federal University of Sergipe, São Cristóvão, Brazil; 5 Department of Pharmacy, Federal University of Sergipe, Lagarto, Brazil

**Keywords:** Transgender persons, Health services, Patient satisfaction, Quality of life, Quality of life, Outcomes research

## Abstract

Trans people face numerous barriers to access and permanence in health services, which makes it difficult, among other things, to know about their quality of life and satisfaction with health services related to these users. Thus, the objective was to describle the quality of life and satisfaction with pharmacotherapeutic follow-up in transgender people. A cross-sectional, descriptive, and quantitative study was conducted between January and September 2022 at a specialized outpatient clinic for transgender individuals. The following aspects were describle: sociodemographic and medication profiles; quality of Life, which was measured using the WHOQOL-BREF questionnaire; and levels of satisfaction with Pharmacotherapeutic Monitoring, assessed through the Pharmacy Services Satisfaction Questionnaire (QSSF). Descriptive analyses employed measures of central tendency, absolute and relative frequencies, while inferential analyses used the Student’s *t* test. A total of 101 transgender individuals participated in the study, with a mean age of 25 years, the majority being single (79.2%/80), having more than 11 years of education (47.5%/n = 48), and comprising 48.5% (n = 49) transgender women. Hormone use was reported by 59.4% (n = 60) of the participants, with 18.3% (n = 11) of self-medication. Testosterone cypionate was the most common hormone used by transgender men (84%), while cyproterone acetate and estradiol represented 60.4% of hormone use among transgender women. Additionally, 36 transgender individuals were taking other drugs (n = 60), mainly antidepressives (28%). The WHOQOL-BREF showed higher scores in the domains of self-rated Quality of Life, and physical, and psychological well-being among transgender women compared to transgender men, but without statistical differences. Income revealed a statistically significant association with psychological domains and overall quality of life. The results of the QSSF indicated that the overall mean and average scores per question were higher than 4, suggesting that 100% of the sample was satisfied with the provided service. No statistically significant differences were observed in the Quality of Life between transgender men and women, but income was associated with the psychological domain and overall Quality of Life. All participants reported satisfaction with the Pharmacotherapeutic Monitoring service; however, there is a need to expand service offerings, such as medication dispensing.

## Introduction

Transgender identity can be understood as a condition where individuals perceive, accept, and allow themselves to assume a gender identity that does not conform to culturally defined norms based on biological characteristics after birth^1^. Thus, transgender identity is understood when there is a discrepancy between biological sex and the various social standards and parameters of gender^2,3^. In this perspective, transgender individuals experience an internal conflict regarding self-perception and the desire for physical adjustments and adaptations that align with their gender identity. In this context, different authors have observed a relationship between transgender identity and quality of life, with key factors being dissatisfaction with vocal self-perception and voice quality^4^, as well as body image concerns^5^.

Other social determinants of health (SDH) that negatively influence the health status of this population group are described in the literature, such as life expectancy. In Brazil, life expectancy for transgender individuals is estimated at 35 years of age, approximately half the national average^6^. Some studies report the prevalence of conditions like binge eating and excessive exercise to meet gender expectations more quickly, resulting in the term known as "passing”^7^. Undesirable outcomes related to hormone use are also described, including insulin resistance, a higher incidence of breast cancer in trans women, and a disproportionately higher burden of mental illnesses such as depression, anxiety, and suicide^8^.

From this perspective, other important considerations about SDH in the transgender population include, for example, gender. Boonyapisomparn et al. observed that transgender women had higher probabilities of accessing and utilizing non-conventional healthcare, such as undergoing procedures in inadequate environments and hormone usage without medical prescription, when compared to transgender men^9^.

In addition to the often-lethal transphobic violence due to the social context in which the Brazilian transgender population is situated, there are other SDH that are of great importance and need to be identified. However, limitations in access to and utilization of healthcare services lead to negative outcomes in the health-disease binomial, resulting in increased morbidity and mortality among these individuals. Thus, it is recognized that various nuances affect access to and utilization of healthcare services, such as the absence or difficulties in accessing specialized services, neglect of the health status of transgender individuals due to transphobia, as well as disrespectful professional attitudes that delegitimize gender identity, even treating transgender identity as a disease^10^. For example, Ho Chan & Suen report that less than 6% of the interviewed trans person mentioned that healthcare providers asked about their sexual orientation. Furthermore, they cite that 8% of the surveyed individuals reported negative experiences in traditional care settings^11^.

The different adversities in accessing and utilizing healthcare services primarily result in the informal, insecure, and irrational seeking of procedures and pharmacological treatments by this population group. Hormone prescription in Brazil is only performed by medical professionals; however, difficulties in accessing and acquiring medications lead to self-medication, which is a prevalent practice among the transgender population^3^.

Recognizing the health needs of this population, both in terms of morbidity and mortality as well as pharmacotherapy aspects and considering the absence of therapeutic protocols and clinical guidelines (PCDT), it is important to note that pharmacists are qualified and suitable professionals for managing pharmacotherapy. The importance of these professionals lies in their involvement in the development of PCDTs, as well as the identification, validation, and management of adverse drug events (ADEs), and the promotion of rational and safe drug use. Thus, the inclusion of this professional category in specialized services for the care of this population allows for the expansion, strengthening, and optimization of processes involved in pharmacotherapy, which significantly impact the quality of life of transgender individuals. This includes the technical management of medication, ensuring the acquisition of appropriate and safe drugs, as well as the clinical management in providing guidance on usage and resolving doubts regarding pharmacotherapy^12^.

In epidemiological studies of care and health promotion, indicators of quality of life and satisfaction with healthcare services have been extensively investigated. These indicators allow for a more rigorous understanding and analysis of important health measurements. Although individual, such analyses allow for the measurement of vital capacities so that quality of life and satisfaction with the service can be compared and evaluate from the perspective of care and health promotion^13–15^.

The WHOQOL-BREF strongly emphasizes the individual's perspective on their own quality of life. This can be particularly useful when focusing on subjective perceptions and personal experiences. This instrument makes it possible to assess the quality of life in different groups and situations, regardless of the level of education. Presenting satisfactory psychometric properties and requiring little application time, the WHOQOL brief makes it possible to describe an individual’s subjective perception of their physical and psychological health, social relationships, and the environment in which they live. From this perspective, the WHOQOL-BREF—validated in the Brazilian population—allows us to visualize this population's vulnerabilities through domains that precisely cover the most sensitive aspects of this population^16^. Considering this context, the present study aimed to describle the Quality of Life and satisfaction with Pharmacotherapeutic Monitoring in transgender individuals receiving care at a specialized clinic in the transgender process in northeastern Brazil.

## Methods

### Study context

It was a cross-sectional, descriptive, quantitative study conducted at a specialized outpatient clinic for transgender individuals from January to September 2022. This service is the first and currently the only one in the state of Sergipe, Brazil, to offer transgender healthcare at the outpatient level. Currently, it operates with the support of the Federal University of Sergipe (UFS) and the Brazilian Hospital Services Company (EBSERH). The service is staffed by a multidisciplinary team of ten specialties, including Endocrinology, Gynecology, Psychiatry, Nutrition, Speech Therapy, Pharmacy, Psychology, Occupational Therapy, Social Assistance, and Nursing.

The consultations are always held on Thursdays in the afternoon, with approximately 153 active users during the research period. The study aimed to assess the sociodemographic, medication, and quality of life profiles of all the clinic's users, as well as evaluate the satisfaction of users receiving Pharmacotherapeutic Monitoring services.

### Participants

The population consisted of transgender individuals (trans men and trans women) and travesties (a term used in the Brazilian context for individuals who adopt feminine gender roles but do not identify as men or women)^17^.

#### Inclusion criteria

This study included users followed at the trans outpatient clinic aged 18 years or over, with or without hormonal treatment, who were treated by any specialty.

#### Exclusion criteria

Given that the quality of life assessment was carried out on the entire population of trans people who attended the outpatient clinic, regardless of specialty, users invited to participate in the research but who did not agree to the Consent Form were excluded from the assessment. Free and Informed (TCLE).

For satisfaction with the Pharmacotherapeutic Follow-up service, trans people who were scheduled but did not attend the Pharmacotherapeutic Follow-up service were excluded from the study, as were users who did not agree to the Informed Consent Form (TCLE).

### Collection and procedures

Transgender individuals were individually attended to by the clinic's team of pharmacists, which consisted of two pharmacy residents from the multi-professional residency program at the University Hospital of Lagarto—Brazilian Hospital Services Company (HUL-EBSERH), two master's students from the Graduate Program in Applied Health Sciences, and one faculty member from the Pharmacy Department of the Federal University of Sergipe, Lagarto Campus (UFS/Lag). Initially, a survey was conducted to collect the sociodemographic and medication profiles of all users who attended the clinic from January to September 2022.

#### Sociodemographic profile

The investigated variables regarding sociodemographic characteristics included gender identity, age group, education level (collected in completed years of study), marital status (classified according to the Brazilian Institute of Geography and Statistics—IBGE), and income (reported and classified according to the prevailing minimum wage at the time: < 1212.00  > 1212.00, or preferred not to disclose). During the survey of participants' sociodemographic profiles, only income data were not included due to the limited completion of medical records and patient forms. Therefore, this variable was collected from July to September 2022, along with other general information, in the quality of life questionnaire.

#### Medication profile

In the pharmacy records and pharmaceutical anamnesis forms, the following information regarding pharmacotherapy was collected: names of prescribed and non-prescribed medications (self-medication), pharmaceutical form, dosage (dose and frequency), and therapeutic indication. Based on this data, the relative frequency was used to better understand hormone use among trans women and trans men.

#### Quality of life

Concurrently, from July to September 2022, the level of quality of life was assessed for all users attending the clinic. The WHOQOL-BREF (World Health Organization Quality of Life)^18^, comprises a self-report instrument consisting of 26 items. Out of these, 24 items assess four domains: physical, psychological, social relationships, and environment, while two items assess the individual's overall self-assessment of quality of life. Each item of the WHOQOL-BREF was scored on a Likert scale from 1 (very dissatisfied/very poor) to 5 (very satisfied/very good).

Since there is no suggestive cutoff point for the better or worse quality of life, this instrument provides a global score and domain scores, represented by mean scores, indicating the individual's perceived satisfaction with each aspect of quality of life. A higher score indicates a better quality of life. The participants were asked to consider the past two weeks when responding to the instrument. For the analysis of this questionnaire, the Microsoft Excel tool developed and suggested by Pedroso et al.^19^ was used^18^.

#### Level of satisfaction with the pharmacotherapeutic monitoring service

From July to September 2022, the level of user satisfaction with the Pharmacotherapeutic Monitoring service was collected and evaluated. Immediately after the consultation, to avoid bias in responses, the interviewing pharmacists who were not present during the consultations administered the Pharmacy Services Satisfaction Questionnaire (QSSF) in a separate environment (in vacant offices and the waiting room). It was determined that a minimum of one consultation was required for the administration of the satisfaction questionnaire, as one of the study limitations was the users' return for continued care.

This instrument was initially developed by Schommer and Kucukarslan^20^ was adapted, translated, and validated in the Portuguese language by Correr et al.^21^. The questionnaire is divided into two blocks of questions. The first block consists of eight questions related to appearance, quality of service, availability, and quality of pharmacist responses, the relationship between professionals and patients, and the courtesy and respect shown by all staff. In the first block, question number 5 ("Promptness in processing your prescription?") was excluded from our questionnaire because medication dispensation services are not currently provided at the clinic.

The second block, consisting of twelve items, refers to the services provided by pharmacists and their performance in patient guidance and responsibility, offered interest, problem-solving, quality of guidance, the privacy of care, and time devoted to patients. In these satisfaction blocks, a Likert-type five-point intensity scale was applied to each question (a total of 19 questions due to the exclusion of one item), with the lowest number representing the option "very poor" and the highest representing "very good." Users were classified as satisfied if their score was equal to or greater than 4 and dissatisfied if it was less than 4.

### Statistical analysis

Microsoft Excel version 2019 was used for data tabulation and descriptive analysis. A descriptive analysis of quantitative variables was performed using measures of central tendency (mean, standard deviation, and median). Relative and absolute frequencies were calculated for qualitative nominal variables. To analyze the associations between sociodemographic profile and quality of life, the Student’s *t *test was used, with the Biostat version 5.3 statistical package.

### Ethical issues

This research met the criteria of Resolution No. 466 of the National Health Council of December 12, 2012, which deals with the guidelines and regulatory standards for research involving human beings, and which is based on the Declaration of Helsinki. The present study received approval from the Research Ethics Committee of the Federal University of Sergipe, UFS Lag/HUL (CAAE: 57407322.0.0000.0217) under Opinion 5,452,695. Before starting the study, users were informed about the objectives and voluntary character of the project, its main aspects and, in case of consent, they signed the Informed Consent Form. It is worth mentioning that the secrecy of the information obtained and the anonymity of the evaluated participants were guaranteed. It is noteworthy that all participants included in this research signed the free and informed consent form, and all data presented in this research were obtained through the informed consent of all participants.

### Ethics approval and consent to participate

The present study received approval from the Research Ethics Committee of the Federal University of Sergipe, UFSLag/HUL (CAAE: 57407322.0.0000.0217) under Opinion 5.452.695.

## Results

### Sociodemographic profile

Between January and September 2022, 101 transgender individuals attended the transgender outpatient clinic at least once. Of this total, 48.5% (n = 49) identified as transgender women, 47.5% (n = 48) as transgender men, 3.0% (n = 3) as travesties, and 1% (n = 1) did not provide information (Table 1). Regarding age, a young sample was observed, as 78.2% (n = 79) of the users were aged 29 years or younger. The mean age was 27.1 years (SD ± 6.2 years), ranging from the youngest at 18 years old to the oldest at 46 years old. It was noted that 79.2% (n = 80) reported being single, and 47.5% (n = 48) had more than 11 years of education (Table 1).

**Table 1:** Distribution of sociodemographic data of transgender individuals treated at a specialized outpatient clinic (n = 101).

Variables	n	%
Gender (n = 101)		
Trans woman	49	48.5
Trans man	48	47.5
Travesti	3	3.0
Not specified	1	1.0
Age group		
18 to 29 years old	79	78.2
≥ 30 years old	22	21.8
Marital status		
Single	80	79.2
Married (a)	6	5.9
Common-law marriage	2	2.0
Divorced	2	2.0
Not specified	11	10.9
Educational level		
< 11 years of education	13	12.9
> 11 years of education	48	47.5
Completed undergraduate education	11	10.9
Postgraduate education	2	2.0
Not specified	27	26.7

Descriptive analysis. n = absolute frequency; % = relative frequency; *Trans* transgender. Own elaboration with data obtained from the research. Lagarto, 2022.

### Medication profile

Regarding the medication profile of the users (Table 2), 59.4% (n = 60) were undergoing hormone therapy during the study period. When asked about self-medication, 18.3% (n = 11) of these users reported using hormones without a medical prescription. As for the most prescribed hormones, overall, testosterone cypionate accounted for 84% (n = 21) of prescriptions for trans men, being the preferred hormonal pharmacotherapy for the development of male characteristics. Conversely, for trans women, the consumption of cyproterone acetate and estradiol accounted for 60.4% (n = 32) of the preferred hormonal pharmacotherapy for acquiring female characteristics.

**Table 2:** Pharmacotherapeutic profile of transgender individuals treated at a specialized outpatient clinic (n = 101).

Variables	n	%
In use of hormonal pharmacotherapy (n = 101)		
Yes	60	59.4
Not	28	27.7
Not specified	13	12.9
Self-medication (n = 60)		
Yes	11	18.3
Not	45	75.0
Not specified	4	6.7
Hormonal pharmacotherapy trans man		
Testosterone cypionate	21	84
Testosterone undecanoate	2	8
Did not provide the information	2	8
Trans woman		
Cyproterone acetate	17	32.1
Estradiol	15	28.3
Estradiol valerate	6	11.3
Algestone acetophenide + estradiol enanthate	6	11.3
Estradiol valerate + levonorgestrel	1	1.9
Nomegestrol acetate	1	1.9
Medroxyprogesterone acetate	1	1.9
Did not provide the information	6	11.3
Use of hormone therapy and other medications		
Yes	36	60
Not	24	40

Descriptive analysis. n = absolute frequency; % = relative frequency; *Trans* transgender. Own elaboration with data obtained from the research.

Lagarto, 2022.

Another point observed regarding the medication profile of these users is that 60.0% (n = 36) of trans individuals, in addition to hormonal pharmacotherapy, reported using other classes of medications in their routine. The main representative class was antidepressants (28.0%), followed by antipsychotics (13.3%), anxiolytics, and benzodiazepines (9.3%).

Using a cross-sectional design from July to September 2022, we applied the WHOQOL-BREF questionnaire to assess Quality of Life and the QSSF instrument to assess Satisfaction with the Pharmacy Service (Pharmacotherapeutic Monitoring). The Quality of Life instrument was applied to all people who attended the outpatient clinic during this period (June to September) and agreed to participate in the research (n = 38), and, of this total, 21 users were also treated by the health service-pharmacotherapeutic Monitoring, where the level of Satisfaction was assessed.

### Quality of life

Table 3 presents the scores of the items and factor scores for the quality of life outcomes of the participants (n = 38) from July to September 2022. In Table 4, it can be observed that trans women had higher scores in the self-assessment, physical, and psychological domains of quality of life, respectively, compared to trans men, but without statistically significant differences (p > 0.05). In both groups, the lowest quality of life score was recorded in the environment domain (11.66 ± 3.00 for trans women and 12.36 ± 3.17 for trans men).

**Table 3:** Measures for items and scores of the WHOQOL-BREF questionnaire factors in transgender individuals (n = 38).

Variables	Media	SD
1. How would you rate your quality of life?	3.47	0.98
2. How satisfied are you with your health?	3.53	1.06
3. What extent do you think your physical pain prevents you from doing what you need to do?	2.05	1.09
4. How much do you need medical treatment to carry out your daily life?	3.45	1.20
5. How much do you enjoy your life?	3.45	1.13
6. What extent do you feel that your life has meaning?	3.70	1.18
7. How well can you concentrate?	3.11	1.13
8. How safe do you feel in your daily life?	3.21	1.14
9. How healthy is your physical environment (climate, noise, pollution, amenites)?	3.16	1.00
10. Do you have enough energy for your daily life?	3.41	1.12
11. Are you able to accept your physical appearance?	2.97	1.35
12. Do you have enough money to meet your needs?	2.13	0.88
13. How readily available is the information you need in your daily life?	3.29	1.14
14. To what extent do you have leisure activity opportunities?	3.00	1.23
15. How well are you able to move around?	4.03	0.91
16. how satisfied are you with your sleep?	2.87	1.14
17. How satisfied are you with your ability to perform daily activities?	3.42	1.11
18. How satisfied are you with your ability to work?	3.24	1.32
19. How satisfied are you with yourself?	3.26	1.13
20. How satisfied are you with your personal relationships (friends, relatives, acquaintances, colleagues)?	3.45	1.11
21. How satisfied are you with your sexual life?	3.61	1.17
22. How satisfied are you with the support you receive from your friends?	3.89	1.16
23. How satisfied are you with the conditions of the place you live in?	2.32	1.25
24. How satisfied are you with your access to healthcare services?	2.95	1.23
25. How satisfied are you with your means of transportation?	3.92	1.34
26. How often do you experience negative feelings such as bad mood, despair, anxiety, depression?	3.21	1.32
Physical domain	13.38	2.79
Psychological domain	12.82	2.91
Social domain	14.60	3.37
Environment domain	11.99	3.06
Self-assessment of quality of life	14.00	3.57
WHOQOL-bref total	13.01	2.51

*SD* standard deviation. Own elaboration with data obtained from the research. Lagarto, 2022.

**Table 4:** Association between quality of life domains in the sample of genders.

	Average and standard deviation			F
	Trans woman	Trans man	p-value	
D. physical	13.52 ± 3.02	13.2 ± 2.73	0.83	1.08
D. psychological	13.21 ± 3.58	12.51 ± 2.41	0.66	1.40
D. social relationships	13.83 ± 3.54	14.92 ± 3.09	0.49	1.23
D. environment	11.66 ± 3.00	12.36 ± 3.17	0.67	1.00
Self-assessment of QV	15.13 ± 3.50	13.05 ± 3.50	0.22	1.15

Lagarto, 2022.

Table 5 demonstrates the association between sociodemographic variables and quality of life scores. A statistically significant association was observed between the two income groups in the psychological domain. The t-value (− 2.50) is significant, with a p-value of 0.0157 (two-tailed), indicating a difference in the psychological domain among the income groups in the sample, with a better perception of this domain among those belonging to an income higher than the minimum wage. Another statistically significant association was observed between the two income groups in the overall aspects of the WHOQOL-BREF. The t-value (− 21.02) is significant, with a p-value of 0.04 (two-tailed), indicating a better overall perception of quality of life among those belonging to an income higher than the minimum wage.

**Table 5:** Association between sociodemographic variables and quality of life domains in the sample (n = 38).

Sociodemographic variables	Physical		Psychological		Social relationship		Enverinonment		Self-assessment of QV		QV overall	
	Average	p	Average	p	Average	p	Average	p	Average	p	Average	P
Income												
< 1 minimum wage	12.22	0.052	11.47	0.015*	13.87	0.313	10.77	0.064	13.07	0.203	11.86	0.04*
≥ 1 minimum wage	14.74		14.58		15.29		13.4		14.93		14.37	
Education												
< 11 years of study	12.6	0.484	12.5	0.785	15.33	0.562	10.75	0.318	12.5	0.274	12.3	0.502
11 years or more of study	13.47		12.86		14.51		12.13		14.18		13.1	
Age range												
18 a 29 years old	13.48	0.762	12.57	0.444	14.8	0.549	12.17	0.597	14.28	0.429	13.08	0.222
≥ 30 years old	13.06		13.63		13.93		11.39		13.11		12.79	

*p < 0.05. Lagarto, 2022.

### Level of satisfaction with the pharmacotherapeutic follow-up service

For a total of 21 trans individuals who responded to the QSSF questionnaire, 38% (n = 8) marked "excellent" for all questions. It is worth noting that the maximum rating (excellent) was given in 84.9% of the questionnaire items. The overall average of all responses for all items was 4.82 ± 0.16, with the lowest averages obtained for questions 13 (4.52 ± 0.85), 18 (4.55 ± 0.74), and 9 (4.74 ± 0.64), respectively, which are specifically related to the pharmacist's practice. This is because, at the time of questionnaire administration, some of these users were still about to start hormonal pharmacotherapy and therefore could not assess the assistance of the professional in these aspects.

Therefore, as the overall average and the average score for all questions were higher than 4, it is considered that 100% of the sample was satisfied with the pharmacotherapeutic monitoring service.

## Discussion

It is worth noting that the gender identity profile found (Table 1) aligns with the majority of findings working with this population and indicates that a significant portion of the sample consists of transgender women, as observed in a study conducted in Brasilia, Brazil by Krüger et al.^22^ where 54.5% identified as transgender women, followed by 30.4% identifying as travesties^22^. Among these studies, a survey of 1788 transgender individuals in the city of São Paulo, Brazil, conducted in 2021, revealed that 43% of this population were transgender women, followed by travesties and transgender men, whose rates were identical (23%).

Regarding education, the data from the present study resemble the findings of a study that assessed the quality of life in a sample of 42 transgender individuals in Iran, which showed that 50.5% of these individuals had completed elementary education^23^. Similarly, in line with previous results, the survey of 1788 transgender individuals conducted in São Paulo, Brazil, indicated that over half of the interviewed population (51%) had completed high school. This scenario of low education, lack of professional qualifications, and the requirements and prejudices faced in various spheres of society pose serious restrictions for the transgender population in accessing the job market^24^

In terms of age, the median in the present study was 25 years. Previous studies in the literature have also reinforced these results, as seen in works conducted in several Brazilian states by Martins et al.^25^ with 304 participants, where half of the sample was under 24 years old. In the study by Grinsztejn et al.^26^ with a sample of 345 participants, the median age was 28 years. In the study conducted by Krüger et al.^22^ with transgender women and travesties, the median age was 24 years. It is observed that studies conducted with transgender individuals predominantly involve young people, with the sociodemographic profile of older age groups remaining unknown.

These facts raise important questions, as the results of these different studies indicate that there are limitations in accessing and utilizing healthcare services for restricted segments of the population, such as older transgender individuals. It is necessary to understand what happens to the population that does not have access to these services and the reasons why they do not use them. In addition to these factors, it is worth noting the low life expectancy of transgender individuals.

In this context, the life expectancy for transgender populations in Brazil is on average 35 years, whereas, for the general population in 2013, the average was 74.9 years. According to these authors, the main causes of this high mortality rate are transphobic violence, HIV/AIDS infection, and clandestine medical interventions^26^.

Regarding hormone use, it was observed that more than half of the transgender individuals were using these medications, and a significant portion of these users self-medicate with hormones without a medical prescription. In the study by Silva et al.^27^ with 127 travesties and transgender women, 94.8% reported hormone use, and of these, 68.1% mentioned the practice of self-medication.

It is worth noting that although the previously mentioned study by Silva et al.^27^ is almost equivalent to the present study in terms of the number of participants, their sample focused only on transgender women and travesties, which may explain the high prevalence of hormone use. While feminizing hormones require a medical prescription, they are more easily obtained compared to androgens. Additionally, the scarcity of qualified professionals who can prescribe these medications more appropriately and safely, as well as the quality of access and utilization of medical services, also contribute to this situation.

Regarding hormone therapy, the most used hormone for transgender men was testosterone cypionate, an androgen hormone. On the other hand, transgender women mostly used cyproterone acetate (an anti-androgen) and estradiol (an estrogen hormone). This profile is similar to the findings in the study conducted by Augusto et al.^10^ which investigated the most commonly prescribed hormones in seven specialized healthcare facilities for transgender individuals in a state in southern Brazil. Except for one healthcare facility, all seven facilities prescribed cyproterone acetate, testosterone cypionate, and estrogen medications, with only variations in the route of administration.

Furthermore, among the other mentioned medications, antidepressants were the most commonly used in this study. These findings align with a study conducted in a city in southern Brazil, where 77% of transgender individuals reported using psychotropic drugs, particularly selective serotonin reuptake inhibitors (SSRIs) as the most commonly used medication for depressive symptoms^27^. Difficulties in access, discrimination, and unmet expectations in public healthcare are among the contributing factors to the development of depression.

In assessing the quality of life of the transgender population, there are few documented studies on this subject in Brazil, while international studies are also scarce, as most existing studies on the quality of life of transgender individuals are related to the outcomes of gender-affirming surgeries^23,28,29^. However, the available studies on quality of life and the differences between transgender men and transgender women often yield contrasting results, which can be attributed to the heterogeneity of the sample and the different stages of treatment, leading to methodological limitations^30^.

Moreover, in this study, the results obtained with the WHOQOL-BREF demonstrate that transgender women had higher scores in three domains: self-rated quality of life, physical, and psychological. In contrast, in the study by Motmans et al.^31^ which involved 148 transgender individuals, transgender women had lower quality of life scores compared to transgender men in the physical and general health scales.

Furthermore, based on the perspective provided by Bartolucci et al.^32^ under exacerbating discriminatory factors, transgender individuals have a lower quality of life compared to the general population, especially in the physical and social domains. More than half of the patients also reported unsatisfactory sexual life^7^.

According to a study conducted with 71 transgender individuals using the Short Form-36 (SF-36) questionnaire, which also assesses the quality of life, it was evident that transgender women had lower quality of life scores than transgender men only in the physical domain. Similarly, transgender individuals with a higher socioeconomic level experience a better quality of life compared to those with lower education and socioeconomic status, who encounter more difficulties such as challenges in maintaining treatment due to its high cost, which can severely affect the quality of life, particularly in terms of mental health^29^.

Therefore, reflecting on the results found by Valashany & Janghorbani^30^ regarding sociodemographic variables, the numbers obtained in this study replicate this pattern: users with income below the minimum wage and those with low education levels consistently responded with lower averages in almost all items. This factor is relevant as it explores the complexity of the social dimension as a source of inequities, thus fostering another form of violence^32^.

Although the scores from the WHOQOL-BREF do not function as a tool for diagnosing mental or physical health, low scores in specific domains, such as environmental quality of life, can allow planners and healthcare providers to better assess what type of interventions (e.g. free transportation to specialized clinics) may be necessary at the community program level^33^.

In the present study, no statistically significant association was observed regarding gender between the groups (transgender women and transgender men) concerning quality of life scores. These results are similar to those found by Gorin-Lazard et al.^34^, Auer et al.^35^, Jellestad et al.^36^, when analyzing the quality of life among transfeminine and transmasculine individuals.

Therefore, what can make a significant difference is the surrounding environment. It is the improved housing conditions, employment, education, higher socioeconomic status, and therapeutic interventions that act as predictors of greater psychological well-being and are associated with better quality of life for these individuals^29^. Thus, in this study, income among sociodemographic factors presented a statistically significant association, with the perception that psychological and general quality of life domains are better with higher income^36^.

Considering the above, when considering a pharmaceutical therapy monitoring service in a transgender clinic, it is natural that, due to remnants of the biomedical model, the role of the pharmacist is centered around the provision and dispensing of medications, especially hormones. However, contrary to common belief and shifting away from a medication-centered approach, the service encompassed in this study is organized with a focus on the individual, with the use of medication as a link to care^37,38^.

When measuring the level of satisfaction of transgender users with the pharmacotherapeutic monitoring service, an overall high average was perceived in the evaluation, which may indicate, according to the analysis of participants' responses, that the provided care is adequate. However, the lack of a pharmacy that supplies and dispenses medications and hormones through this service was pointed out as the main problem to be resolved. According to Bandeira et al.^39^ there is a relationship between access to medications and satisfaction with healthcare services, with higher satisfaction among participants who have access to all the medications they need. However, as the questionnaires were administered within the ambulatory settings, users may have omitted more information due to insecurity or fear of not being served.

Although the results shown in this study were derived from closed-ended questionnaires, some users felt comfortable expressing their opinions and suggestions regarding the pharmacotherapeutic monitoring service, and the main suggestion was related to the need for medication and hormone dispensing by the pharmacy.

Despite these limiting factors, when comparing the results obtained in this study with the frequency of "excellent" responses assigned to each item of the questionnaire, a score of 84.9% maximum rating was achieved. In other studies that assessed satisfaction with pharmacy services in an integrated manner with other populations, such as Correr et al.^21^ the frequency of "excellent" responses was only 36.1%, and in the study by Custódio^40^, the frequency of "excellent" responses was 53%.

Upon investigating the questions with the highest ratings, the present study obtained higher scores in items 20, 2, 6, 7, 14, 15, and 16, with question 20 "The amount of time the pharmacist spends with you?" receiving the highest average rating of 4.95. In other words, the highest ratings attributed to satisfaction were related to aspects of clinical care provided by the pharmacist, and the average duration of these consultations ranged from 30 to 40 min. In Custódio’s study^40^, the questions with the highest ratings were items 1 and 14, both with an average of 4.92. In the study conducted by Larson et al.^41^, questions related to the quality of instructions, the professional's ability to provide guidance, and the pharmacist's availability to clarify doubts obtained the highest scores.

Undoubtedly, assessments of patient satisfaction with healthcare services, specifically pharmaceutical services, are valuable because satisfied patients are more likely to have a better relationship with the attending professional, greater interest in their health, and consequently, better outcomes. Given the findings, it is crucial for healthcare services to adopt measures that promote the improvement of services provided in order to achieve greater user satisfaction^42^.

Satisfaction evaluations by the transgender population regarding healthcare services are still underexplored, but some research has been conducted on the assessment of specialized services in gender reassignment, which evaluated patients' subjective satisfaction after surgery^43^. However, there are currently no records in the databases indexing health studies regarding satisfaction with a specific Pharmacotherapeutic Monitoring service.

As described by Cocohoba^44^ pharmacists can promote inclusion and acceptance of transgender individuals, counsel patients on the effects of hormone therapy, and provide essential preventive healthcare services such as immunizations and screenings for Human Immunodeficiency Virus (HIV). In the present study, no relationships were identified between satisfaction results and patient profiles in terms of age, gender identity, or education level. For a more comprehensive evaluation of these aspects, it is suggested that further studies be conducted involving a larger population.

### Limitations

Despite the efforts made to apply questionnaires on quality of life and satisfaction levels, it was not possible to expand the sample, even after several attempts throughout the study period (3 months), mainly due to the specificity of the population. In this situation, due to the small sample size, statistical differences were not observed in the scores of the quality of life domains according to gender. Still, two statistically significant associations were observed between the two income groups with the psychological domain and those with the general aspects of the WHOQOL-BREF, indicating a better public perception of quality of life among those with income above one minimum wage. Another significant limitation was evaluating the Pharmacotherapeutic Monitoring service, as we did not determine, in the case of users with more than one consultation, at which point the service would be evaluated; thus, these users may have reported their best or worst query. Individuals not included in the analysis of quality of life and level of satisfaction have similar sociodemographic and clinical characteristics to those included. The difference between the two subgroups was the month of service. However, since the clinical conditions of individuals are not influenced by seasonality, this difference, although necessary, may not impact the results observed. Still, we suggest that future studies watch and avoid such discrepancies. In addition to these facts, there are numerous barriers and weaknesses in the specialized clinic itself, such as, for example, the location of the health facility in the interior region of the state, approximately 88 km from the capital; its operation, which occurs only one day a week, in a single shift, every two weeks; medication is not dispensed (especially hormone therapy); Another determining factor is the lack of promotion of the clinic among the transgender community and health professionals in the network, as there are people in the municipality who are unaware of the services offered.

## Conclusion

Considering the observed aspects, the sociodemographic and medication profile revealed a predominantly young sample, mostly consisting of transgender women, mostly single, with more than 11 years of education, most of them using hormonal pharmacotherapy, and approximately 20% through self-medication. Regarding the investigation of Quality of Life, only income significantly impacted the psychological domain and overall quality of life of these individuals.

The satisfaction level of users with the Pharmaceutical Therapy Monitoring service was satisfactory, especially regarding the clinical monitoring provided by healthcare professionals. However, the lack of medication dispensing was spontaneously identified by users as a primary issue to be addressed. These results highlight the need to identify the sociodemographic and pharmacotherapeutic profile in older individuals and understand the possible limitations regarding access and utilization of these healthcare services for restricted segments of the population. It is necessary to understand what happens to the population that does not have access and the reasons why they do not use these services.

Given the classification of the satisfaction level of the Pharmaceutical Therapy Monitoring service and the perception of Quality of Life, efforts should be made to improve accessibility to healthcare services, as it is now considered a critical component in determining the quality of life not only for the individual but for society. However, there is still a scarcity of studies with robust evidence regarding the health of the transgender population worldwide and in Brazil. The lack of data on this population is mainly due to their exclusion from demographic censuses. In this scenario and considering the importance of this social segment, this work will contribute to a better characterization of this population so that public agencies and organizations can obtain more information about its diversity.

## Data Availability

Data supporting the findings of this study are available from Carla Maria Lima Silva. However, there are restrictions on the availability of these data, which were used under license for the current study and are therefore not publicly available. However, the authors make the data available upon reasonable request and with the authorization of Carla Maria Lima Silva.

## Abbreviations

SDH: Social determinants of health
PCDT: Therapeutic protocols and clinical guidelines
QSSF: Pharmacy services satisfaction questionnaire
